# Exploring the Effect of Red and Blue on Cognitive Task Performances

**DOI:** 10.3389/fpsyg.2016.00784

**Published:** 2016-05-26

**Authors:** Tiansheng Xia, Lu Song, Ting T. Wang, Ling Tan, Lei Mo

**Affiliations:** ^1^School of Psychology and Center for Studies of Psychological Application, South China Normal UniversityGuangzhou, China; ^2^School of Nursing, Guangzhou University of Chinese MedicineGuangzhou, China; ^3^Research Center for Psychology and Special Education, National Institute of Education SciencesBeijing, China

**Keywords:** color effect, red, blue, approach/avoidance motive, arousal

## Abstract

Previous studies on the effect of color on cognitive task performances and have led to two different views. Some researchers think that the influence of red and blue on cognitive tasks is modulated by the difficulty of the task, and other researchers suggest that the influence mainly depends on the type of task. The current study combined these factors to investigate the effect of color on cognitive task performance. In Experiment 1, we manipulated the difficulty of the task to investigate the effect of red and blue on detail-oriented task performance (the proofreading task), whereas in Experiment 2 we manipulated task difficulty to explore the effect of red and blue on creative task performance (the Remote Associates Test). The results showed that red enhanced the performance on a simple detail-oriented task. However, blue improved the performance on a difficult detail-oriented task as well as on both simple and difficult creative tasks. The results of the current study indicate that the type and difficulty of the task together modulate the effect of color on cognitive performances.

Color is ubiquitous and plays an important role in individuals’ experience of the world (Elliot et al., 2007). Humans encounter a world that is rife with color; color is present in our dreams. Color has fascinated scholars for a long time and color research looks beyond color esthetics to the link between color and psychological functioning in humans (Elliot and Maier, 2014; Elliot, 2015).

The psychological effect of color has been widely investigated, with most studies focusing on the colors red and blue (Mehta and Zhu, 2009) because they are two of the three primary colors, and because red is a long wave color and blue is a short wave color. Red and blue both are of representative in the physical sense. Some studies have suggested that red enhances task performances as compared with blue (Stone, 2003; Elliot and Aarts, 2011; Zhang and Han, 2014); others have found exactly the opposite results (Elliot et al., 2007). For example, Kwallek and Lewis (1990) found that participants made fewer errors in a red office than in a white office. Another study also indicated that when performing a low demand task (making a written record about two reservation sessions based on audiotapes), participants’ performances were better in the red than blue environment (Stone, 2003**).** In contrast, other research has suggested that red should undermine performance on challenging intellectual tasks (Soldat et al., 1997; Elliot et al., 2007; Shi et al., 2015; Thorstenson, 2015). Elliot et al. (2007) found that compared with green and white, brief perception of red prior to two cognitive tests (i.e., an anagram test and an IQ test) impaired performance.

The influences of color on cognition and behavior are seen as a result of learned associations (Elliot et al., 2007). In daily life, some situations in the context of different colors are often accompanied by particular experiences. When repeatedly encountering these situations, people can form specific associations between colors and these experiences and/or concepts (Mehta and Zhu, 2009). For example, red is commonly associated with danger and mistakes (e.g., incorrect answers are marked in red ink); by contrast, blue is commonly associated with openness and peace (e. g., the sky and the sea are both blue) (Elliot et al., 2007; Gerend and Sias, 2009).

Mehta and Zhu (2009) further proposed that red and blue color can induce alternative motivations through different associations related to red and blue. Specifically, red should cause an avoidance motivation due to its link with dangers and mistakes, which made individuals pay more attention to avoid making mistakes and falling into the danger. Thus, red should enhance performance on detail-oriented tasks (e.g., text proofreading and recall tasks that require focused, careful attention). By contrast, blue should induce an approach motivation because blue is often related to openness and peace, which signal a benign environment that encourages individuals to use innovative strategies in a more explorative manner. Hence, blue, as compared to red, should enhance performance on creative tasks (e.g., word associations, IQ tests and tests of analogical reasoning, which assess divergent thinking and memory resources). This view is also documented in other research (Tanaka and Tokuno, 2011).

Elliot and Maier (2012) further integrated previous studies and proposed the color-in-context theory, which draws on social learning theory and the field of biology. This theory indicates that color has multiple associations and effects that differ as a function of context. Context can include domain (e.g., red has positive associations in the attraction domain but negative associations in the achievement domain such as cognitive task performance) and type of task (e.g., red has positive effects on low-level, detail oriented tasks, but negative effects on high-level tasks that require mental manipulation) (Elliot and Maier, 2014; Elliot, 2015).

However, the studies mentioned above may ignore one important factor, namely the difficulty of tasks. Being different from Mehta and Zhu (2009) who emphasized the role of the different motivations induced by red and blue in different types of cognitive task, other studies found that depending on the level of difficulty, the same color could produce different levels of performances on the same type of cognitive task. For example, Soldat et al. (1997) found that for the same type of mathematical analysis tasks, when the test items were more difficult, the participants’ accuracy was higher in the blue context than in the red context; conversely, when the test items were easier, the participants’ accuracy in the blue and red contexts was not significantly different. Stone (2003) also found that when completing less difficult written record, the red context was more beneficial than other color contexts for completing the tasks, and with increasingly difficult tasks, the performance in the red context was worse. That is, the influence of red and blue on cognitive tasks may be modulated by the difficulty of the tasks. One possible reason for this phenomenon is the relationship between color and emotional arousal.

A widely accepted view is that long-wave colors (e.g., red) can cause higher arousal than short-wave colors (e.g., blue) (Wexner, 1954; Wright and Rainwater, 1962; Jacobs and Suess, 1975; Walters et al., 1982; Stone, 2003). Wexner (1954) assessed participants’ subjective evaluation and found that red was related to the adjective “excited,” and blue was related to the adjective “calm.” Jacobs and Hustmyer (1974) tested the skin electric responses of participants under different light environments, and the results showed that participants had the highest arousal in the red context, followed by green, yellow and blue. Walters et al. (1982) argued that the arousal associated with color was related to the color’s position in the spectrum: long-wave colors (e.g., red, orange, yellow) are the colors of arousal, short-wave colors (e.g., blue, indigo, and purple) are the relaxed colors, and green in the middle position is the neutral color. Bagchi and Cheema (2013) also documented that red (vs. blue) background elicited higher jumps in auction bidding, presumably because the red color induced aggression through arousal.

Although the relationship between color and emotional arousal is still under debate (Wright, 1998; Fehrman and Fehrman, 2004; Elliot et al., 2007), the classic theory of the relationship between the difficulty of tasks and emotional arousal, named the Yerkes–Dodson law, provides one possible framework for conceptualizing this relationship (Yerkes and Dodson, 1908; Diamond et al., 2007). According to the Yerkes–Dodson law, when simple tasks are manipulated, high arousal (related to red) is more advantageous than low arousal (related to blue); in contrast, when difficult tasks are manipulated, low arousal (related to blue) is more advantageous than high arousal (related to red).

On this basis, we suggest that the type and difficulty of tasks both modulate the color effect via motivation and arousal, respectively. Integrating the two lines of research (the associations between color and motivation and between color and arousal), the current study puts forward basic ideas on the influence of red and blue on cognitive task performance: for a *simple detail-oriented task*, red, as compared to blue, has a positive role in motivation and arousal; in contrast, for a *difficult creative task*, blue, as compared to red, has a positive role in motivation and arousal. However, for a *difficult detail-oriented task*, according to the hypothesis of color-motivation associations, red has advantage in motivation and thus can enhance task performance, whereas blue has an advantage in arousal according to the hypothesis of color-arousal associations and thus blue can enhance task performance; similarly, for the *simple creative task*, red has advantage in arousal, whereas blue has advantage in motivation. Hence, for the effect of color on *simple detail-oriented task* and *difficult creative task* performances, we can expect their results, whereas the effect of color on *difficult detail-oriented task* and *simple creative task* performances was an exploratory question in the present study.

To further explore the role of color on the cognitive task performances, the current study investigated the influence of red and blue on performance on different types of cognitive tasks (e.g., detail-oriented tasks and creative tasks) with different difficulty levels (i.e., simple and difficult), and tested the difference due to the motivation and arousal caused by red and blue. We employed the tasks used by Mehta and Zhu (2009), that is, a text proofreading task (detail-oriented task) and a remote association task (creative task) in the current experiment. The setup criteria of red and blue were same as those in Mehta and Zhu’s (2009) research, although we used gray rather than white as the baseline color. This was because gray could be consistent with red and blue in the brightness dimension and thus was more rigorous than white as a control, see **Table 1**.

**Table 1:** Properties of red, blue and gray.

	HSL criterion				RGB criterion		
	Red	Blue	Gray		Red	Blue	Gray
Hue	0	160	0	R	255	0	120
Saturation	240	240	0	G	0	0	120
Lightness	120	120	120	B	0	255	120

## Experiment 1

Following research by Mehta and Zhu (2009), which suggested that the color red enhances cognitive task performance compared with blue, this experiment further investigated the influence of red and blue on detail-oriented task performance at different levels of task difficulty.

### Method

#### Participants

A total of 125 college students were recruited from Guangzhou, China (ages 18–25, 93 females), and they were divided randomly into three experimental groups. Each group completed the detail-oriented task with a different colored background: red, blue, or gray. According to their self-report, no participants had color-blindness, color weakness, or other vision disorders. All participants had normal or corrected to normal vision, which refers to having visual acuity above 1.0, as measured by an eye chart (Legge et al., 1985). The study protocol was approved by the South China Normal University Research Ethics Committee, and it was performed in accordance with the ethical standards specified in the 1964 Declaration of Helsinki and its later amendments. Written informed consent was obtained from each participant, and participants could freely withdraw from the study at any time.

#### Materials and Procedure

We adopted the same proofreading task as Mehta and Zhu (2009), which is generally used to test individuals’ attention to detail. Participants were instructed to examine 20 sets of items, each containing a pair of names or addresses that were either identical or slightly different. Participants were instructed to judge whether items within each pair were identical or not. The 20 sets were divided evenly into two groups: the simple group had fewer letters (20–50) and the difficult group had more letters (50–100). A pilot test showed that judgment accuracy was significantly higher for sets in the simple group compared to the difficult group, *t*(19) = 4.00, *p* = 0.001. An example of one set **is** as follows: “*Jean-Philippe 876van Dijck*”; “*Jean-Philipe 876van Dijck.*” Each pair was presented in the center of the screen, with a vertical line between items.

We adopted a 3 (Color: red, blue, gray) × 2 (Task difficulty: difficult, simple) mixed-design, with color as a between-subjects variable and task difficulty as a within-subjects variable. E-Prime 2.0 (Microsoft Inc.) was used to program the task, present stimuli, and record behavioral responses in the experiment. Participants sat in front of the monitor at a viewing distance of about 60 cm and completed the task alone in a soundproof room. The screen was set as follows: brightness = 70, contrast = 80, and color temperature = 6500K.

The 20 pairs of alphabetic strings were randomly displayed to the participants on a specific color background (i.e., red, blue, or gray) based upon condition. Participants were asked to judge whether items within each pair were identical or not (e.g., order of letters, position, capitalization, punctuation). Participants responded via key press, after which the next pair would appear on the screen. There was no time limit for responses; however, participants were instructed to respond to the stimulus as quickly and accurately as possible.

#### Statistical Analysis

To test the effect of red and blue colored backgrounds on performance on a detail-oriented task at simple and difficult levels, we performed a 3 × 2 mixed-design analysis of variance (ANOVA), using color (three levels: red, blue, gray) as the between-subjects factor, task difficulty (two levels: difficult, simple) as the within-subjects factor, and task accuracy rate as the dependent variable.

### Results and Discussion

**FIGURE 1:**
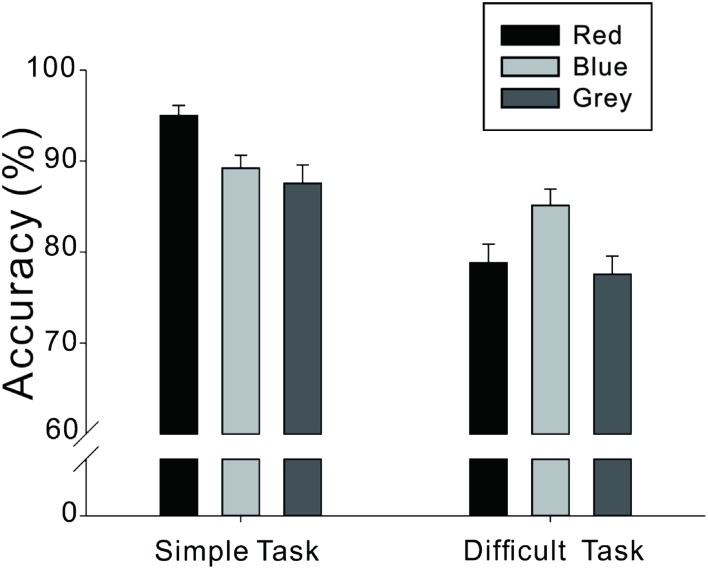
**Accuracy (%) on detail-oriented tasks of different levels of difficulty and under different color backgrounds**.

The data of three participants were removed as their error rate was above 80%, which were out of the range of 3 standard deviations from the mean of each condition. As illustrated in **Figure 1** and **Tables 2** and **3**, the ANOVA revealed a significant main effect of task difficulty, *F*(1,119) = 63.38, *p* < 0.001, and a significant main effect of color, *F*(2,119) = 3.10, *p* < 0.05. Additionally, there was a significant interaction between task difficulty and color, *F*(2,119) = 7.54, *p* < 0.01. Further analysis indicated that the differences between red, blue, and gray were significant within the simple task condition, *F*(2,119) = 5.93, *p* < 0.01, suggesting that the accuracy rate of those in the red background condition was higher than the accuracy rate of those in the blue background condition, *t*(79) = 3.10, *p* < 0.01, and the gray background condition, *t*(81) = 3.14, *p* < 0.01. There was no difference between those in the blue and gray background conditions, *t*(78) = 0.65, *p* > 0.05. There were also significant differences between red, blue, and gray backgrounds within the difficult task condition, *F*(2,119) = 3.90, *p* < 0.05. However, in contrast to those in the simple task condition, the results showed that the accuracy rate of participants in the blue background condition was higher than the accuracy rate of those in the red background condition, *t*(79) = -2.21, *p* < 0.05, and the gray background condition, *t*(78) = 2.69, *p* < 0.01. There was no difference in accuracy between the red and gray background conditions, *t*(81) = 0.67, *p* > 0.05. In addition, we performed a similar 3 × 2 mixed-design ANOVA for the reaction times. The results revealed a significant main effect of task difficulty, *F*(1,119) = 765.70, *p* < 0.001. However, there were no significant main effect of color, *F*(1,119) = 1.41, *p* = 0.249, and no significant interaction effect between task difficulty and color, *F*(2,119) = 2.03, *p* = 0.137, suggesting the absence of speed-accuracy tradeoff.

**Table 2:** Accuracy (%) on detail-oriented and creative tasks with different levels of difficulty.

	Red	Blue	Gray
	*M (SD)*	*M (SD)*	*M (SD)*
**Detail-oriented task**			
Simple	95.0 (7.4)	89.2 (9.3)	87.6 (13.4)
Difficult	78.8 (13.7)	85.1 (11.9)	77.6 (13.2)
**Creative task**			
Simple	74.3 (12.3)	82.3 (13.1)	73.7 (18.0)
Difficult	31.1 (13.7)	38.1 (14.4)	31.9 (16.4)

**Table 3:** Results of ANOVA analysis.

		Sum of squares	*df*	Mean square	*F*	*p*	$\eta_{p}^{2}$
Detail-oriented task	Difficulty	62.14	1	62.14	63.38	0.000	0.348
	Color	10.94	2	5.47	3.10	0.049	0.050
	Interaction	14.79	2	7.40	7.54	0.001	0.113
Creative task	Difficulty	751.61	1	757.61	420.97	0.000	0.844
	Color	19.68	2	9.84	0.381	0.026	0.089
	Interaction	0.38	2	0.19	0.11	0.900	0.003

Results of the simple detail-oriented task was consistent with our hypothesis, suggesting that when completing the simple detail-oriented task, the red background was more beneficial than the blue background; in contrast, when completing the difficult detail-oriented task, the blue background was more beneficial than the red background, while no such difference was found between the red and gray background, which suggested that the positive effect from avoidance motivation induced by red was offset by the negative effect from high arousal in the difficult task. In addition, these results also indicated that, no matter whether the detailed-oriented task was simple or difficult, there were no significant differences between the disadvantageous color background and the gray background as the baseline. That is, it appears that background color had either a “facilitating effect” or “no facilitating effect,” but it did not have an “interference effect” on the cognitive task performances.

## Experiment 2

Following research by Mehta and Zhu (2009), which suggested that the color blue enhances creative task performance compared to the color red, this experiment further investigated the influence of red and blue on creative task performance at different levels of task difficulty.

### Method

#### Participants

A total of 81 college students were recruited from Guangzhou, China (ages 18–25, 56 females), and they were divided randomly into three experimental groups. Each group completed the same creative task with a different colored background: red, blue, or gray. No participants reported color-blindness, color weakness, or other vision disorders. All participants had normal or corrected to normal vision.

#### Materials and Procedure

Following Mehta and Zhu (2009), we adopted the same Remote Associates Test (RAT) as the creative task. Compared with other creative tasks, the RAT has several advantages, such as simple operation, objective scoring, unique answer, and brief administration time (Bowden and Jung-Beeman, 2003). In this test, each question was composed of three words, and the target answer was a single word that could form a compound word with each one of the previous three words. For example, the target answer for “shelf, worm, end” is “book” (with the three compound words being “bookshelf, bookworm, bookend”). Given the familiarity of language, we created a version of the RAT in Chinese. In this version, each question was composed of three Chinese characters, and the target answer was a Chinese character that could form common words with each one of the previous three Chinese characters. For example, “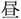, 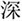, 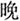
” (English equivalent: day, deep, evening) had the target answer of “
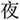
” which refers to night in English (they separately form “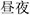, 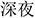, 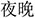
” [English equivalent: day and night, late at night, night]).

We created 120 test items and recruited 75 participants (about half of them were liberal arts students) to assess these materials. Using their responses, 21 test items were removed from the final test because they had more than one correct answer. From the remaining items, we selected 10 easier test items (>0.7) and 10 more difficult test items (<0.4), based on the rate of accuracy for each item. These 20 test items were used as the materials in Experiment 2.

We adopted a 3 (Color: red, blue, gray) × 2 (Task difficulty: difficult, simple) mixed-design, with color as a between-subjects variable and task difficulty as a within-subjects variable. E-Prime 2.0 (Microsoft Inc.) was used to program the task, present stimuli, and record behavioral responses in the experiment. Participants sat in front of the monitor at a viewing distance of about 60 cm and completed the task alone in a soundproof room. The screen was set as follows, brightness = 70, contrast = 80, and color temperature = 6500K.

The 20 test items were displayed one at a time in a random order to the participants on a specific color background (i.e., red, blue, or gray), with each participant only ever seeing one background color. Participants were instructed to report the answer as quickly and accurately as possible, and they were given 30 s for each item. The SoundIn control of E-Prime 2.0 was used to record participants’ answers, and the experimenters counted the accuracy rate of each participant according to the recordings. After participants reported their answer for a test item, the next item appeared on the screen.

#### Statistical Analysis

To test the effect of red and blue colored backgrounds on performance on a creative task at simple and difficult levels, we performed a 3 × 2 mixed-design ANOVA, using color (three levels: red, blue, gray) as the between-subjects factor, task difficulty (two levels: difficult, simple) as the within-subjects factor, and accuracy rate as the dependent variable.

### Results and Discussion

**FIGURE 2:**
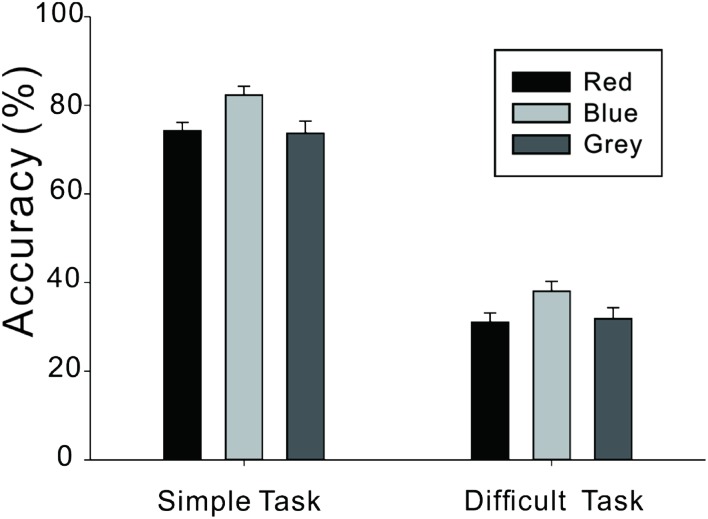
**Accuracy (%) on creative tasks of different levels of difficulty and under different color backgrounds**.

As illustrated in **Figure 2** and **Table 2**, the ANOVA revealed a significant main effect of task difficulty, *F*(1,78) = 420.97, *p* < 0.001. There was also a significant main effect of color, *F*(2,78) = 3.81, *p* < 0.05, indicating that accuracy rate was significantly higher for those in the blue background condition than both those in the red background condition, *p* < 0.05, and those in the gray background condition, *p* < 0.05. There was no difference between those in the red and gray background conditions, *p* > 0.05. The interaction between task difficulty and color was not significant, *F*(2,78) = 0.11, *p* > 0.05.

Result of the difficult creative task was consistent with our hypothesis. The blue rather than the red enhanced the performance in simple creative task, suggesting that the high arousal induced by red hardly has any influence on creative task whereas the positive effect from the approach motivation induced by blue remained valid even in the simple creative task. Hence, these results indicated that regardless of creative task difficulty, the blue background enhanced participants’ performance compared to the red background. In addition, these results also indicated that there was no significant difference between the disadvantageous color background (red) and the gray background as the baseline. As in Experiment 1, these results suggested that color had either a “facilitating effect” or “no facilitating effect,” but it did not have an “interference effect” on cognitive task performance.

## General Discussion

The results of Experiment 1 showed that under the easy detail-oriented tasks condition, the red context was more advantageous than the blue context; by contrast, for difficult detail-oriented tasks, the blue context was more advantageous than the red context. The results of Experiment 2 indicated that for creative tasks, whether or not the task was difficult, the blue context was more advantageous than the red context. These results suggest that the factors of task type and task difficulty collectively modulated the influence of color on cognitive task performances.

The results were neither completely consistent with the expectation based on color-motivation associations, nor completely consistent with the expectation based on color-arousal associations, implying that there was an interaction between color-motivation and color-arousal associations. The results are in agreement with those of Stone (2003), perhaps in part because both studies used similar task as a detail-oriented task. Both studies found that when performing a low demand (simple) task, performance appeared to worsen over time in the blue rather than the red environment; in contrast, when working on a high demand (difficult) task in a red environment, performance was worse. In another study, Soldat et al. (1997) instructed participants to complete both simple and complex problem solving tasks on either red or blue paper. The results indicated that for complex tasks, blue paper led to better performance than did in red paper; however, no difference was apparent for simple tasks. The problem solving tasks were regarded as a creative task, and hence the findings seem to be contradictory to the results in the present experiment. However, one possible explanation is that the simple problem solving task in their study was too simple, resulting in a ceiling effect.

Elliot et al. (2007) posited that red is associated with failure and danger and evokes avoidance motivation in achievement contexts, and Mehta and Zhu (2009) suggested that blue is associated with openness and peace and evokes approach motivation. Avoidance motivation is regarded as facilitating performance on basic, detail-oriented cognitive tasks that require minimal mental manipulation or flexibility (Friedman and Förster, 2010), and, accordingly, some researchers offered and found evidence to support the proposal that red facilitates performance on such tasks (Mehta and Zhu, 2009; Elliot and Aarts, 2011; Sorokowski and Szmajke, 2011).

However, some factors, beyond task type, also warrant consideration as boundary conditions and moderators of the influence of red on cognitive performance (Elliot and Maier, 2014). For example, the red effect may vary as a function of the difficulty level of the task, which is consistent with the results in the present study. Given color-arousal associations, the Yerkes–Dodson law posited that high arousal (related to red) facilitates performance on simple tasks and undermines performance on difficult tasks (Yerkes and Dodson, 1908; Diamond et al., 2007). However, the influence of color on creative tasks in the present study did not correspond with our expectations based on the Yerkes–Dodson law. One possible explanation is that creative tasks need more innovative thinking and an explorative approach rather than focused, careful attention, and hence they are usually regarded as relatively more difficult tasks, as compared to the detail-oriented tasks.

Though we did not investigate when and how emotional arousal and motivation were related to color in cognitive tasks in the present study, we have used functional magnetic resonance imaging (fMRI) to explore this issue in another study. The results indicated that when performing the detail-oriented task, red activated avoidance motivation-related brain regions (left fusiform gyrus), as well as negative emotional brain areas (left parahippocampal gyrus); in contrast, when performing the creative tasks, blue activated approach motivation-related brain areas (left frontal cortex). No matter whether in the detail-oriented tasks or creative tasks, the emotional arousal caused by red was higher than blue (i.e., greater activation in the visual cortex).

## Conclusion

Our studies found a different influence of three color backgrounds (red, blue and gray) on two types of cognitive tasks (detail-oriented task and creative task) with two levels of difficulties (simple or difficult). That is, red enhanced performance on a simple detail-oriented task. However, blue improved performance on a difficult detail-oriented task as well as on creative tasks, no matter whether the task was simple or difficult.

## Author Contributions

LM developed the study concept. All authors contributed to the study design. Testing and data collection were performed by LS and TW performed the data analysis. TX and LS wrote the paper. TX and LT revised this manuscript. All authors approved the final version of the paper for submission.

## Conflict of Interest Statement

The authors declare that the research was conducted in the absence of any commercial or financial relationships that could be construed as a potential conflict of interest.
